# Experimental evidence for stabilizing selection on virulence in a bacterial pathogen

**DOI:** 10.1002/evl3.203

**Published:** 2020-11-11

**Authors:** Camille Bonneaud, Luc Tardy, Geoffrey E. Hill, Kevin J. McGraw, Alastair J. Wilson, Mathieu Giraudeau

**Affiliations:** ^1^ Centre for Ecology and Conservation, Biosciences University of Exeter Penryn Cornwall TR10 9FE United Kingdom; ^2^ Department of Biological Sciences Auburn University Auburn Alabama 36849‐5414, United States of America; ^3^ School of Life Sciences Arizona State University Tempe Arizona 85287‐4501, United States of America

**Keywords:** Emerging infectious disease, house finches, *Mycoplasma gallisepticum*, replication rate, transmission rate

## Abstract

The virulence‐transmission trade‐off hypothesis has provided a dominant theoretical basis for predicting pathogen virulence evolution, but empirical tests are rare, particularly at pathogen emergence. The central prediction of this hypothesis is that pathogen fitness is maximized at intermediate virulence due to a trade‐off between infection duration and transmission rate. However, obtaining sufficient numbers of pathogen isolates of contrasting virulence to test the shape of relationships between key pathogen traits, and doing so without the confounds of evolved host protective immunity (as expected at emergence), is challenging. Here, we inoculated 55 isolates of the bacterial pathogen, *Mycoplasma gallisepticum*, into non‐resistant house finches (*Haemorhous mexicanus*) from populations that have never been exposed to the disease. Isolates were collected over a 20‐year period from outbreak in disease‐exposed populations of house finches and vary markedly in virulence. We found a positive linear relationship between pathogen virulence and transmission rate to an uninfected sentinel, supporting the core assumption of the trade‐off hypothesis. Further, in support of the key prediction, there was no evidence for directional selection on a quantitative proxy of pathogen virulence and, instead, isolates of intermediate virulence were fittest. Surprisingly, however, the positive relationship between virulence and transmission rate was not underpinned by variation in pathogen load or replication rate as is commonly assumed. Our results indicate that selection favors pathogens of intermediate virulence at disease emergence in a novel host species, even when virulence and transmission are not linked to pathogen load.

**Impact Summary**

With increasing threats of emerging infectious diseases, there is a pressing need to understand why novel pathogens harm their hosts and how their virulence is expected to evolve over time. One of the few ways of understanding virulence evolution at pathogen emergence is to inoculate individuals of the novel host species with pathogen variants of differing virulence and investigate their ensuing success. We conducted such an infection experiment in a North American songbird using 55 distinct variants of its emerging infectious bacterial pathogen that differed in virulence. To ensure that we measured pathogen variant success in conditions equivalent to an epidemic outbreak in a novel host species, we caught all birds from populations that had never been exposed to the disease and verified that none had previously been naturally‐infected with the pathogen. We demonstrate that more virulent variants transmitted faster, but had shorter infection durations, leading to variants of intermediate virulence being more evolutionarily successful. Interestingly, we did not find support for the common suggestion that the number of pathogen cells underpins their virulence and transmission rate. Our results provide much needed empirical support for the dominant theoretical understanding of pathogen success and show that, at epidemic outbreak in a novel host species, selection will favor pathogen variants of intermediate virulence.

The virulence‐transmission trade‐off hypothesis constitutes a central theoretical foundation for understanding pathogen fitness (Anderson & May 1982; Ewald 1983; Alizon et al. 2009; Alizon & Michalakis 2015). Under this hypothesis, virulence, which is defined in mathematical models as pathogen‐induced host mortality rate (Read 1994; Alizon & Michalakis 2015), is predicted to evolve toward an intermediate optimum. This is because the exploitation of the host, which is necessary for transmission, also increases host mortality rate; thereby reducing the time window for successful transmission. Thus, the trade‐off between transmission rate and infection duration is expected to lead to stabilizing selection on pathogen virulence. While there is growing support for the assumption that virulence and transmission are positively and directly associated (Dwyer *et al*. 1990; Mackinnon & Read 1999; Mackinnon *et al*. 2002; Mackinnon & Read 2003; Mackinnon *et al*. 2008; Chapuis *et al*. 2012; Doumayrou *et al*. 2013; Williams *et al*. 2014; Kerr *et al*. 2015; Ben‐Ami 2017), demonstrations of the prediction that selection acts against ever‐increasing virulence and instead favors an intermediate optimum, are rarer (e.g., Jensen *et al*. 2006; Fraser *et al*. 2007; de Roode *et al*. 2008; Doumayrou *et al*. 2013; Read *et al*. 2015), particularly at pathogen emergence in novel hosts.

The relative paucity of empirical demonstrations of selection for intermediate virulence stems from limited numbers of distinct pathogen isolates used in experiments (Doumayrou *et al*. 2013; Williams *et al*. 2014; Acevedo *et al*. 2019), coupled with the challenges of measuring transmission and fitness throughout the life cycle of the pathogen (Jensen *et al*. 2006; de Roode *et al*. 2008; Cressler *et al*. 2016; Acevedo *et al*. 2019). For example, Jensen et al. (2006) used two clones of *Pasteuria ramosa* to show that *Daphnia magna* hosts with intermediate longevity had the greatest number of pathogen spores, suggesting that this pathogen was most fit at intermediate levels of virulence. Whether there is a monotonic positive relationship between pathogen load and transmission success, or whether there are, instead, threshold effects on the numbers required to transmit (de Roode *et al*. 2008) or trade‐offs between replication rate and longevity (Walther & Ewald 2004; Boonekamp *et al*. 2014) remains to be clarified. Using direct measures of transmission, Williams et al. (2014) showed a positive association between virulence and transmission rate across 3 isolates of the bacterial pathogen, *Mycoplasma gallisepticum*, inoculated into groups of house finches (*Haemorhous mexicanus*); although the relationship between virulence and fitness was not investigated. Read et al. (2015), on the other hand, showed that the two most virulent strains of Marek's disease virus (of the 5 tested) only transmitted when inoculated to chicken that had been vaccinated, suggesting selection against high virulence in nonvaccinated hosts. Finally, using 20 isolates of the vector‐borne insect bacterial pathogen, *Xenorhabdus nematophila*, Chapuis et al. (2012) provided evidence of a trade‐off between bacterial load and fitness, but a linear positive relationship between virulence and fitness (not the expected intermediate fitness optimum).

One way to further our understanding of the shape of the association between virulence and fitness is to measure virulence and keyfitness components (including transmission) in a sufficient number of pathogen isolates to adopt a regression‐based approach (Inouye 2001; Gotelli & Ellison 2004; Williams *et al*. 2014; Acevedo *et al*. 2019). Here, we attempted to do so in an inoculation experiment of house finches using 55 distinct isolates of their conjunctivitis‐causing bacterial pathogen, *M. gallisepticum*, collected from its emergence (in 1994) and throughout the subsequent 20 years (until 2015) (*N* = 46 isolates successfully established infection in 47 birds). These isolates have been shown previously to vary in virulence, with later‐epidemic isolates causing greater putative host mortality, as well as more severe conjunctival swelling and greater body mass loss than early‐epidemic ones (Tardy *et al*. 2019). Further, while this bacterial pathogen has now spread throughout most of the North American house finch range, some western populations have remained unexposed to present day (Staley et al. 2018a). We can therefore use non‐resistant finches from unexposed populations to test for associations among genetically‐determined pathogen traits, while removing the confounding effects of evolved immune clearance by the host (Mackinnon & Read 2004; Barclay et al. 2012; Kerr et al. 2017). Although hosts from disease‐unexposed populations will vary naturally in their ability to deal with the infection, this variation allows us to investigate the shape of the virulence‐fitness relationship in natural conditions representative of those experienced by the pathogen at outbreak. By randomly allocating hosts to isolates, we ensure that the among‐host variation is randomly distributed across isolates. In addition, by housing each inoculated bird in a cage with an uninfected sentinel, also from disease‐unexposed populations, we are able to obtain a direct measure of transmission capacity, irrespectively of whether it occurs as a result of direct contact or via fomites, and without the confounding ecological effects of variation in the probability that infected individuals encounter conspecifics. Using a single uninfected sentinel, rather than a group, also ensures that transmission arises from the experimentally inoculated individual only and cannot reflect secondary transmission events originating from other sentinels. While necessarily conducted in captivity, our set‐up mimics natural conditions because house finches are highly social, visit both food sources (including feeders) together, and additionally show bi‐parental care, meaning that individuals are typically in close, often physical, contact with conspecifics (Hill 2002).

We have 3 specific aims. First, we test the assumption that virulence and transmission rate are positively associated. Second, we use a regression analysis to investigate the shape of the relationship between a quantitative proxy of virulence and fitness. Third, we test the commonly proposed hypothesis that variation in pathogen load (or replication rate) underpins the association between virulence and transmission rate (Anderson & May 1982; Ewald 1983; Frank 1996; Ebert 1998; Gandon & Michalakis 2000; Gandon *et al*. 2001; Ferguson *et al*. 2003; Jensen *et al*. 2006; Fraser *et al*. 2007; Little *et al*. 2008). In house finches, *M. gallisepticum* colonizes the mucosal surfaces of the conjunctiva and upper respiratory tract, causing severe conjunctivitis. These symptoms are essential for transmission, which occurs through eye secretions transferred directly or left on inert surfaces as fomites that have been shown to persist for a maximum of 24 h (see Methods) (Dhondt *et al*. 2007). In severe cases, the infection can lead to mortality in the wild when house finches become lethargic and visually‐impaired, thereby becoming unable to feed or escape predation (Roberts *et al*. 2001a; Adelman *et al*. 2017) – indeed millions are suspected to have died in this way within years of outbreak in 1994 (Dhondt *et al*. 1998; Nolan *et al*. 1998). As a consequence, we measured putative host mortality as the loss of avoidance response to hand capture (Kollias *et al*. 2004). Because this behavioral change is associated with severe symptoms of conjunctivitis (Roberts *et al*. 2001b; Kollias *et al*. 2004; Adelman *et al*. 2017) (see Methods), we used the mean extent of conjunctival swelling over the course of the experiment (measured from photographs) as a quantitative proxy for virulence in our analyses. Transmission rate to the uninfected sentinel was measured every 1–3 days by PCR amplification of *M. gallisepticum* DNA collected from conjunctival and tracheal swabs (Roberts *et al*. 2001a). Fitness was measured as the product of infection duration in the experimentally inoculated host (in days to loss of avoidance response to hand capture) and transmission rate to the uninfected sentinel (in days^−1^) (Anderson & May 1979; Alizon & Michalakis 2015).

## Methods

### CAPTURE AND HOUSING

Wild house finches from populations that are outside the current range of *M. gallisepticum*, and have thus not evolved genetic resistance, were captured in variety of urban and suburban sites in Arizona during summer 2015 (*N* = 118, 64 males and 54 females). *M. gallisepticum* has never been recorded in the sampling area despite continuous monitoring (Staley *et al*. 2018a). Using birds that have not evolved protective immune responses to *M. gallisepticum* is essential for measuring pathogen load, replication rate, virulence and transmission rate without the confounds of the evolved capacity for immune clearance (Kerr *et al*. 2017) – and no birds cleared the infection during the experiment. Finches that had hatched in spring 2015 were retained, weighed, and banded with a numbered metal tag for individual identification. They were then immediately transported by car to indoor cages (0.40 × 0.29 × 0.21 m) at Arizona State University's Tempe campus, where they were housed for the remainder of the experiment. On arrival, we obtained a blood sample from all birds using brachial venipuncture (60 μL of whole blood) and a choanal swab. A lack of prior infection with *M. gallisepticum* since hatching was confirmed by screening blood plasma for anti‐*M. gallisepticum* antibodies using a serum plate agglutination assay (Luttrell *et al*. 1996), and a lack of current infection was verified using the choanal swabs in PCR amplification of *M. gallisepticum* DNA (Roberts *et al*. 2001b). Although none of the birds displayed any sign of infection with other diseases, all birds were prophylactically medicated for infection with *Trichomonas gallinae* with carnidazole (Spartrix, Janssen/Elanco) and *Isospora* spp. with sulfadimethoxine over 40 days. The birds were then allowed to acclimate for one additional month prior to the experimental onset and provided with *ad libitum* food and water throughout.

### EXPERIMENTAL INOCULATION

The birds were divided into 2 groups of 59 individuals. Each of the 55 *M. gallisepticum* isolates sampled over the course of the epidemic was inoculated into one bird selected at random from the 59 birds of the first group (30 males and 29 females), with 4 isolates (two from 1995 and two from 2007) inoculated in two birds each. Maximizing the number of pathogen isolates used is essential for clarifying the shape of pathogen trait associations in a regression‐based statistical approach (Inouye 2001; Gotelli & Ellison 2004). The alternative of using fewer isolates replicated across multiple hosts would be more appropriate to fully characterize differences among pathogen isolates, but that was not the aim of the study. Isolates were originally obtained from naturally infected, wild‐caught house finches by swabbing the conjunctiva of a symptomatic bird and placing the swab in SP4 growth medium. Isolates were collected at varying times of the year over a 20‐year period, and from various urban and suburban sites in 8 different states in the eastern US (primarily from Alabama). Isolates were administered via 20 μL of culture containing 1 × 10^4^ to 1 × 10^6^ color changing units/mL of *M. gallisepticum* in both eyes. Following inoculation, all 59 birds were maintained in separate cages and co‐housed with one bird from the second group in 54 randomly‐assigned female‐male pairs and 5 male‐male pairs. Birds from the second group thus served as uninfected sentinels (see Introduction for ecological relevance of the set‐up). All birds had access to *ad libitum* food and water throughout the duration of the experiment. The experiment was stopped at 34 dpi and all birds were euthanized at 35 dpi as stipulated by home office licensing. Protocols were approved by Institutional Animal Care and Use Committees (IACUC) of Arizona State University (permit #15‐1438R), as well as by Institutional Biological Use Authorizations to Auburn University (# BUA 500), and the University of Exeter's ethics committee.

### HOST MORTALITY, CONJUNCTIVAL SWELLING, TRANSMISSION RATE, AND FITNESS

Putative host mortality was determined in situ on days 3, 6, 8, 14, 21, 25, 28, and 34 post‐infection, with infection considered lethal when birds lost the avoidance response to hand capture (Roberts *et al*. 2001b; Kollias *et al*. 2004; Adelman *et al*. 2017). This behavioral change is associated with severe signs of conjunctivitis: a red to purple conjunctiva, feather matting extends below the eye or to the entire face with heavy feather loss, eyes difficult to see, and producing excessive discharge, as well as overall depressed motor activity. Such behavioral and physical symptoms, with little or no vision possible and persistent inactivity, are thought to have caused the death of millions of infected finches (Roberts *et al*. 2001b; Kollias *et al*. 2004; Adelman *et al*. 2017). Infection duration was measured as days to putative death or the duration of the experiment + 1 day for isolates that caused sub‐lethal symptoms (i.e., 35 days).

A quantitative proxy for virulence was also obtained from the extent of conjunctival swelling. Measures of conjunctival swelling were obtained by photographing the right and left eyes at 6, 13, 25, and 34 dpi from a standardized distance. We then measured the average area (in pixels) of the conjunctiva swelling across the two eyes and at each day as the area of the outer ring minus the area of the inner ring at 6, 13, 25, or 34 dpi (Staley *et al*. 2018b). Measurements of photographs were done blindly with respect to the identity of the isolate inoculated, and were done independently of (and blindly with respect to) behavioral observations conducted *in situ* during the experiment and used to assess putative host mortality. Measures of conjunctival swelling were taken on multiple days to provide an average across the experiment. The use of a single summary measure (here the mean) simplifies subsequent analyses and their presentation. It particularly provides a justifiable proxy of isolate virulence given the repeatability (at the isolate level) of swelling across time points within hosts. Indeed, using a linear mixed model with days post‐inoculation as a fixed 4‐level factor and isolate as a random effect, we found that among‐isolate differences accounted for 46% of the variation in conjunctival swelling (likelihood ratio test comparison to a reduced model; χ^2^ = 55, *df* = 1, *P* < 0.0001).

Transmission rate to the sentinel was determined by amplification of *M. gallisepticum* DNA from conjunctival and tracheal swabs (Roberts *et al*. 2001a) obtained from the sentinel at 2, 3, 4, 5, 6, 7, 8, 11, 14, 17, 20, 23, 26, 29, 32, and 35 days post‐inoculation (dpi). Our measurement of transmission accounts for the different transmission routes of the pathogen (direct or via fomites) since it allows us to determine the presence of *M. gallisepticum* at the site of infection in the sentinels. The transmission was considered successful when the pathogen was present in the swabs of the sentinel on two consecutive samples and the transmission rate was calculated as 1 divided by the number of days to the first positive swab (in days^−1^). Swabs were taken by gently rolling a sterile flocked swab (moistened in tryptose phosphate broth) once over each eye, and using a separate swab the same procedure was over the choana. This sampling method will inevitably reduce the pathogen load, but this slight reduction will be comparable across all birds (and therefore isolates). It is highly improbable that our swabbing method removed all pathogen cells because it was not conducted over the inner surface of the conjunctiva.

Finally, fitness was calculated as the product of infection duration (in days to putative mortality) and transmission rate (in days^−1^). Isolates obtained a fitness value = 0 when they failed to transmit to the sentinel, and/or when they transmitted only after inducing putative death in the experimentally inoculated host. Because transmission can occur through fomites that are deposited on inert surfaces and that are thought to persist for a maximum of 24 h (Sydenstricker *et al*. 2005), we verified that none of the isolates that transmitted only after inducing putative death in the experimentally inoculated host, did so within 24 h of host death. The minimum amount of time between putative host death and transmission for those isolates was 72 h, indicating that the persistence of fomites will not have impacted our estimates of pathogen fitness.

### PATHOGEN LOAD AND REPLICATION RATE

Bacterial load was measured by quantitative amplification of *M. gallisepticum* DNA from pooled conjunctival and tracheal swabs obtained at 8, 14, 21, and 28 dpi. DNA was extracted using a QIAGEN DNeasy® Blood and Tissue Kit according to the manufacturer's standard protocol. For each sample, we ran a multiplex quantitative PCR of the *M. gallisepticum*‐specific gene *mgc2*, which encodes a cytadhesin protein, and the house finch recombination‐activation gene *rag1*, using an Applied Biosystems™ StepOnePlus™ Real‐Time PCR system (Tardy *et al*. 2019). Each reaction contained: 2 μL of sample genomic DNA template, 1 μL each of 10 μM mgc110‐F/R and rag1‐102‐F/R primers (total 4 μL), 0.5 μL each of 10 μM Mgc110‐JOE and Rag1‐102‐6FAM fluorescent hydrolysis probes (total 1 μl), 10 μl of 2X qPCRBIO Probe Mix HI‐ROX (PCR BIOSYSTEMS) and 5 μL Nuclease‐free water (Ambion^®^). Reactions were then run at 95°C for 3 min, followed by 45 cycles of 95°C for 1 s and 60°C for 20 s. Samples were run in duplicate alongside serial dilutions of plasmid‐based standards (range of standards for *mgc2*: 1.6 × 10^8^ – 1.6 × 10^3^ copies; range of standards for *rag1*: 8.0 × 10^7^ – 8.0 × 10^2^ copies). Amplification data were exported to LinRegPCR version 2017.1 for calculation of individual reaction efficiencies and quantification of low‐amplification samples (Ruijter *et al*. 2009; Tuomi *et al*. 2010); between run variation was normalized using Factor qPCR version 2016.0 (Ruijter *et al*. 2015), with plasmid standard serial dilutions used for factor correction. Pathogen load was then determined as the number of *M. gallisepticum* cells divided by the number of house finch cells to control for variation in sampling efficiency (Grodio *et al*. 2008).

In non‐resistant hosts from disease‐unexposed populations, replication rate can be estimated as pathogen load divided by the time required to reach that load. Because the timing to peak pathogen load has been shown to vary among isolates (Tardy *et al*. 2019), replication rate was measured by dividing peak pathogen load by the number of days to peak load in order to encapsulate the log‐linear phase of the infections. While we acknowledge that growth need not be strictly linear, we fitted time to peak load as a covariate in the model to account for any variation in growth trajectories on the relationship between replication rate and transmission rate.

### STATISTICAL ANALYSES

All statistical analyses were conducted in R version 3.3.2 (R Core Team 2016) and figures were made using ggplot2 (Wickham 2009). Out of the 59 birds experimentally inoculated, 11 failed to become infected and were removed from this study. We removed one additional bird that died during the course of the experiment due to incomplete data. We thus analyzed data from 47 birds inoculated with 46 isolates (one isolate from 2007 was inoculated into two birds). We analyzed the association between transmission rate and putative host mortality or conjunctival swelling using linear models. In both analyses, the response term (transmission rate + 0.1) was natural log‐transformed to ensure normal residuals, and putative mortality or mean conjunctival swelling was fitted as the explanatory term (0.1 was added to all values of transmission rates to allow log‐transformation of four isolates that failed to transmit). In neither analysis was the result altered qualitatively by using mixed model equivalents with year of isolate sampling as a random term (estimate ± SE: putative mortality = 0.45 ± 0.11; mean conjunctival swelling = 0.01 ± 0.003). To analyze the association between fitness and mean conjunctival swelling, we tested for and characterized non‐linear (including stabilizing) selection on conjunctival swelling using a standard spline approach (Schluter 1988), implemented as a general additive model (GAM) in the R library mgcv (Wood 2017). We then verified the lack of directional selection on mean conjunctival swelling by performing a linear model with fitness as the response term, and mean conjunctival swelling as the explanatory term. Finally, to test for associations between pathogen load or replication rate and transmission rate, we ran three linear models with transmission rate (ln transformed) as the response term. As explanatory terms, we fitted: (1) peak pathogen load; (2) total pathogen load calculated as the integral of pathogen load over the course of the 34 day‐experiment; or (3) replication rate (fitting time to peak load as a covariate; see above). We further tested the role of pathogen load (peak and total) and replication rates in driving the association between conjunctival swelling and transmission using a linear model with transmission rate (ln transformed) as the response term and with both mean conjunctival swelling and either peak pathogen load, total pathogen load or replication rate as the explanatory terms (see Supporting Information for further details).

## Results

### ASSOCIATION BETWEEN VIRULENCE AND TRANSMISSION

**Figure 1:**
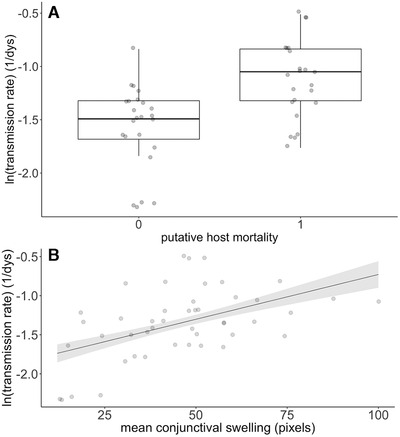
Association between virulence and transmission. We show transmission rate (in day^−1^) as a function of virulence, with virulence measured as: (**A**) putative host mortality based on the loss of avoidance response to hand capture, which occurs as a result of infection‐induced lethargy and blindness; and (**B**) mean conjunctival swelling (in pixels) determined from photographs. Points represent jittered raw values; the lines are predicted from the model with the standard error represented by the ribbon.

The virulence transmission trade‐off hypothesis assumes a positive relationship between virulence and transmission. In support, we found a significant positive association between putative host mortality (i.e., loss of avoidance response to hand capture) and transmission rate (Fig. 1; linear effect model, estimate ± SE = 0.4 ± 0.1, *t*_45_ = 4.0, *P* < 0.0003), with isolates giving rise to lethal behavioural changes transmitting faster to uninfected sentinels than those that did not. Similarly, we found a significant positive relationship between our quantitative proxy for virulence (mean conjunctival swelling) and transmission rate (Fig. 1B; linear effect model, estimate ± SE = 0.01 ± 0.003, *t*_44_ = 3.9, *P* < 0.0004), with pathogen isolates that caused greater mean conjunctival swelling transmitting faster to the uninfected sentinel. A significant positive relationship between conjunctival swelling and transmission rate was also obtained using a bivariate mixed model to estimate the regression of transmission rate on the repeatable (among‐isolate) component of conjunctival swelling measures obtained at 6, 13, 25, and 35 dpi (see Supporting Information).

### TESTING THE PREDICTED ASSOCIATION BETWEEN VIRULENCE AND FITNESS

**Figure 2:**
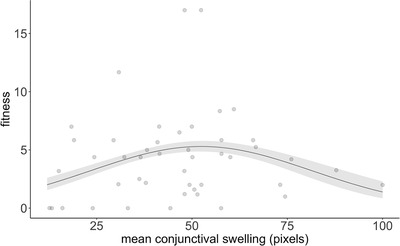
Association between fitness and conjunctival swelling. We show fitness (measured as the product of putative infection duration and transmission rate) as a function of mean conjunctival swelling (in pixels), with greater fitness being associated with intermediate values of mean conjunctival swelling. The two isolates with the highest fitness were collected from two different years; evidence of selection on intermediate values of mean conjunctival swelling remained even when they were removed from the analysis (GAM; parametric linear effect ± se = 0.08 ± 0.009, *t* = 9.2, *P* < 0.0001; spline (non‐linear) effects, F_1.6,2.0_ = 5.8, *P* < 0.006). Points represent jittered raw values; the line is predicted from the model with the standard error represented by the ribbon.

The fundamental prediction of the trade‐off hypothesis is that there is stabilizing selection favoring pathogen isolates of intermediate virulence. We determined pathogen fitness as the product of putative infection duration (measured as the number of days to the loss of the avoidance response to hand capture) and transmission rate to the uninfected sentinel (in days^−1^) (as recommended, Anderson & May 1979; Alizon & Michalakis 2015). We then used nonlinear splines to determine the shape of the virulence‐fitness relationship in a general additive model (GAM). This non‐parametric approach is recommended because stabilizing selection requires that intermediate trait values are most fit, irrespectively of the functional form relating the independent variable to fitness (Schluter 1988). In support of the trade‐off hypothesis, we found evidence of a significant nonlinear relationship between our quantitative proxy for virulence (mean conjunctival swelling) and fitness (GAM; parametric linear effect ± SE = 0.1 ± 0.01, *t* = 7.8, *P* < 0.001; spline (nonlinear) effects, F_1.9,2.3_ = 3.7, *P* < 0.03). Visualizing the model fit confirmed a predicted fitness maximum at an intermediate level of conjunctival swelling (Fig. 2), thus providing evidence of stabilizing selection (note: removing the 2 outlier values of high fitness did not qualitatively affect our results; see legend of Fig. 2). That virulence is not under strong directional selection is further supported by the fact that mean conjunctival swelling is not a statistically significant predictor of fitness in a simple linear regression (linear effects model, slope estimate ± SE = 0.02 ± 0.03, *t*_44_ = 0.5, *P* = 0.6). Finally, since the duration of infection was necessarily right censored for sub‐lethal isolates in which hosts did not reach putative mortality (see Methods), we confirmed that the non‐linear relationship between conjunctival swelling and fitness was robust to assumptions made about the infection duration of sub‐lethal isolates (Figs. S1 and S2). Thus, we found no firm evidence for directional selection on virulence overall, and all our results were consistent with stabilizing selection around an intermediate virulence optimum.

### PATHOGEN LOAD DOES NOT UNDERPIN TRANSMISSION RATE

**Figure 3:**
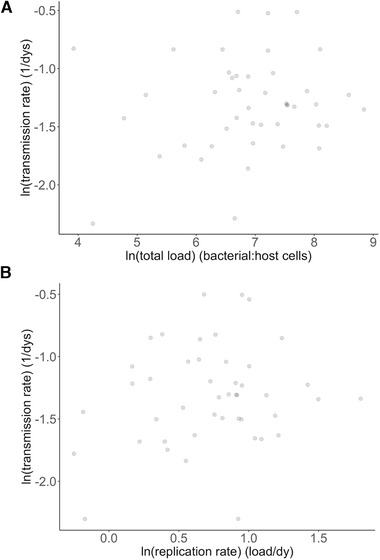
Association between transmission rate and pathogen load. We show transmission rate (in days^−1^) as a function of: (**A**) total pathogen load (ratio of bacterial:host cells) and (**B**) pathogen replication rate (load. day^−1^). Points represent jittered raw values.

Pathogen load (or replication rate) has been shown previously to explain little variance in virulence (measured as putative host mortality, mean conjunctival swelling, body mass loss) in this system (Tardy *et al*. 2019), and here we found no compelling support for the commonly assumed positive association between pathogen load (or replication rate) and transmission rate. Following the exclusion of 2 isolates that failed to replicate during the experiment and showed barely detectable peak loads (see Supporting Information), we found little evidence to suggest that pathogen load predicts transmission rate across the remaining 96% of infections; irrespectively of whether pathogen load was measured as peak load or as total load over the course of the 34 day‐experiment (peak pathogen load: linear model, estimate ± SE = 0.09 ± 0.06, F_1,43_ = 1.5, *P* = 0.1; total pathogen load: linear model, estimate ± se = 0.05 ± 0.06, F_1,42_ = 0.9, *P* = 0.4; Fig. 3A). Further, in non‐resistant hosts from disease‐unexposed populations, replication rate can be estimated as peak pathogen load divided by the time required to reach that load – this is because pathogen load will reflect pathogen replication with no confounding influence of immune clearance. As for pathogen load, we found no significant association between variation in replication rate and transmission rate (Fig. 3B; linear model, estimate ± SE = 0.07 ± 0.07, F_1,42_ = 1.0, *P* = 0.3). This result was not confounded by any among‐isolate variation in the time taken to reach peak load (estimate ± SE = 0.009 ± 0.008, F_1,42_ = 1.3, *P* = 0.3). Finally, pathogen load (peak and total) and replication rate were not found to explain the association between mean conjunctival swelling and transmission rate when they were entered as covariates in the model (Table 1). Thus, although we found support for the prediction of the virulence‐transmission trade‐off hypothesis, our results suggest that pathogen load is not a significant mediating mechanism of increased transmission rate; with implications for using pathogen load as a surrogate for transmission.

**Table 1:** Effect of pathogen load and replication rate in driving the association between mean conjunctival swelling and transmission rate (as in Fig. 1B)

Linear effect model	Explanatory term	Estimate ± SE	*t* Statistics	*P*‐value
1	Conjunctival swelling	0.08 ± 0.003	*t*_41_ = 2.6	0.01
	Peak pathogen load	0.06 ± 0.06	*t*_41_ = 1.0	0.3
2	Conjunctival swelling	0.009 ± 0.003	*t*_40_ = 2.8	0.008
	Total pathogen load	0.03 ± 0.05	*t*_40_ = 0.6	0.6
3	Conjunctival swelling	0.008 ± 0.003	*t*_41_ = 2.8	0.009
	Replication rate	0.04 ± 0.06	*t*_41_ = 0.7	0.5

## Discussion

By conducting experimental inoculations using a large number of isolates of varying virulence of the emerging bacterial pathogen, *M. gallisepticum*, and direct measures of transmission rate over the life cycle of the pathogen, we found support for the virulence‐transmission trade‐off hypothesis in an infectious pathogen at outbreak in a novel host species. First, we showed that isolates that caused putative host mortality and greater mean conjunctival swelling transmitted faster to uninfected sentinels. These results are consistent with the assumption of the trade‐off hypothesis that virulence and transmission rate are positively associated. Second, we found support for the fundamental prediction that fitness estimates are maximized for intermediate levels of a quantitative proxy for virulence (mean conjunctival swelling), indicating that conjunctival swelling was under stabilizing selection. Finally, we found support for the trade‐off hypothesis independently of variation in pathogen load or replication rates, and variation in pathogen load had little or no direct effect on transmission rate. Our results therefore indicate that, at disease emergence, measures of pathogen fitness can be predicted by the virulence‐transmission trade‐off hypothesis, even in this host‐pathogen system in which variation in virulence and transmission are not obviously underpinned by variation in pathogen load.

Despite the central role of the trade‐off hypothesis in our understanding of pathogen fitness, relatively few studies have demonstrated a fitness advantage for pathogens of intermediate virulence (Jensen *et al*. 2006; Fraser *et al*. 2007; de Roode *et al*. 2008; Doumayrou *et al*. 2013; Read *et al*. 2015). As introduced above, there are a number of logistical reasons for this, including the difficulty of measuring all the necessary parameters (Alizon & Michalakis 2015; Cressler et al. 2016), as well as conducting experiments with sufficient numbers of pathogen isolates to adopt the preferred regression‐based approach (Inouye 2001; Gotelli & Ellison 2004; Acevedo *et al*. 2019). While we have been able to overcome some of these challenges, others remain. First, through maximizing the number of isolates used to facilitate the regression‐based approach, we were not able to introduce replicate samples. We attempted to mitigate the lack of replicates by standardizing the host environment through the use of naïve birds from populations that have never been exposed to the pathogen. Nevertheless, we acknowledge that our approach will introduce natural noise, although we have no reason to believe that this should influence the shape the virulence‐fitness relationship since finch hosts were allocated randomly to isolates. Second, while it is relatively straight‐forward to determine infection duration for pathogens that induce host mortality, it is more challenging to do so for pathogens that only cause sub‐lethal infections; particularly in the absence of host immune clearance. Uncertainty over the infection duration of sub‐lethal isolates will affect the gradient of the virulence‐fitness curve during its initial increasing phase, since ∼90% of such isolates are on the left half of the curve (Fig. 2; Fig. S2). Previous evidence has shown that *M. gallisepticum* infection, determined as the presence of clinical signs of conjunctivitis and/or detectable pathogen cells in conjunctival or choanal samples, will last between 27 to 83 days post‐inoculation in non‐resistant house finches (Sydenstricker *et al*. 2005). Whether we censored the infection duration of sub‐lethal isolates to the duration of the experiment + 1 day (i.e., 35 days) or to the maximum duration of infection found in Sydenstricker *et al*. (2005) (i.e., 83 days), the non‐linear relationship between virulence and fitness remains (see Supporting Information and Fig. S2).

The generality of the trade‐off hypothesis has sometimes been questioned on the grounds that, for it to operate, pathogen load needs to underpin virulence and transmission, which is unlikely to be general (Bull 1994; Levin & Bull 1994; Lipsitch & Moxon 1997). For example, when disease symptoms result primarily from host immune responses rather than from pathogen replication (Lipsitch & Moxon 1997), virulence and transmission have been thought to be unlikely to be correlated with each other; undermining the assumption of the trade‐off hypothesis. Nevertheless, this conjecture assumes that variation in virulence plays no role in predicting transmission independently of pathogen load. In *M. gallisepticum*, virulence and pathogen load (or replication rate) are, at best, only weakly correlated, and the evolution of increasing virulence in the face of host resistance has not been associated with an increase in pathogen load (or replication rate) (Tardy *et al*. 2019). This disconnect between virulence and pathogen load is likely to be a consequence of the fact that the amount of harm done by *M. gallisepticum* to its host is largely driven by the pathogen's ability to manipulate the host immune system (Chambaud *et al*. 1999; Szczepanek *et al*. 2010a; Szczepanek *et al*. 2010b; Staley & Bonneaud 2015). Indeed, immune manipulation through the induction of a misdirected inflammatory response underpins the establishment of infection and the transmission of *M. gallisepticum* via eye secretions (Gaunson *et al*. 2000; Lam & DaMassa 2000; d'Hauteville *et al*. 2002; Hornef *et al*. 2002; Ganapathy & Bradbury 2003; Gaunson *et al*. 2006; Dhondt *et al*. 2007; Mohammed *et al*. 2007; Szczepanek *et al*. 2010a; Szczepanek *et al*. 2010b). The fact that we failed to find a significant association between quantitative variation in pathogen load and transmission rate further supports a reduced role of replication rate in shaping pathogen success in this system. And yet, we provide convincing evidence to suggest that pathogen fitness is maximized at intermediate levels of virulence, and so support the idea that the virulence‐transmission trade‐off hypothesis offers a general framework for predicting pathogen success. We therefore hypothesize that selection on intermediate virulence can arise independently of any mediating effects of pathogen load whenever symptoms are integral to transmission success.

Nevertheless, our results raise the question as to why pathogen load may be linked to virulence and transmission in some, but not in other, pathogen‐host systems. Convincing evidence of positive associations between virulence and pathogen load, and between transmission rate and pathogen load, come from a variety of systems (Jensen *et al*. 2006; de Roode *et al*. 2008), but most of these are vector‐borne (e.g., malaria), water‐borne (e.g., *Daphnia*), or sexually transmitted (e.g., HIV) diseases (Ebert 1998; Mackinnon & Read 1999, 2003; Fraser *et al*. 2007; Doumayrou *et al*. 2013). In such cases, the chances of a successful transmission will depend on the probability of a pathogen being picked up by a vector, or of being present in sufficient quantities at the site of contact (which is likely proportional to density). In contrast, the link between pathogen load and transmission might be expected to be reduced when transmission relies intrinsically on the development of disease symptoms (i.e., virulence). Obviously, replication is necessary for all types of infections since without replication there is no pathogen to transmit. However, we hypothesize a reduced association between pathogen load and transmission rate above a general threshold level, when virulence plays the dominant role in transmission success. Our results, therefore, push for a careful consideration of whether virulence is likely to be a side‐consequence or a necessary condition of infection and transmission, before using pathogen load as a proxy for virulence and/or transmission rate.

In conclusion, we show support for both the assumption and the prediction of the virulence‐transmission trade‐off hypothesis, with evidence of a positive linear association between virulence and transmission, and selection on intermediate levels of a quantitative proxy for virulence, respectively. Our results have at least two important implications. First, they indicate that the virulence‐transmission trade‐off hypothesis can capture selection on virulence from outbreak in a bacterium in which transmission rate increases with virulence, but not with pathogen load. Second, our results indicate that, despite potential correlations between within‐host pathogen load and amounts of pathogen deposited as fomites, we cannot equate measures of pathogen load to transmission rate or transmission success. We recommend that further tests of the trade‐off hypothesis attempt to measure key traits directly (particularly transmission), without assuming that pathogen load is a suitable proxy of transmission, and especially in pathogens that require symptoms for transmission.

## AUTHOR CONTRIBUTIONS

C.B. conceived and designed the study. G.E.H., K.J.M., M.G., and C.B. obtained the animals and/or bacterial isolates. M.G., K.J.M., and L.T. conducted the experiments. M.G. analyzed the photographs. L.T. conducted the molecular work. C.B. and A.J.W. analyzed the data. C.B. wrote the paper.

## CONFLICT OF INTEREST

The authors declare no conflict of interest.

## DATA ARCHIVING

Data reported in this paper have been deposited in Dryad Digital Repository (https://doi.org/10.5061/dryad.9cnp5hqgh).

Associate Editor: K. Lythgoe

## Supporting information


**Fig S1**. Number of pathogen cells over the course of the experiment.
**Fig S2**. Association between fitness and conjunctival swelling.
