# Supplementary material for: Experimental evidence for stabilizing selection on virulence in a bacterial pathogen
Source: Evol Lett. 2020 Nov 11;4(6):491–501. doi: 10.1002/evl3.203 (PMC7719545; doi:10.1002/evl3.203)
Supplement: Supplementary file 1 — Fig S1. Number of pathogen cells over the course of the experiment. Fig S2. Association between fitness and conjunctival swelling. [file EVL3-4-491-s001.pdf]

Supporting Information

RESULTS

Association between transmission rate and conjunctival swelling at 6, 13, 25 and 35 dpi

Simple linear regression provided evidence of a positive relationship between our simple proxy of virulence (mean conjunctival swelling) and transmission rate (see main text). We verified this finding using a second approach that fully utilised the repeat observations of host conjunctival swelling at 6, 13, 25 and 34 days post-inoculation (dpi). Specifically, we fitted a bivariate mixed model fitted in ASReml-R version 4 (Butler *et al.* 2017), with transmission rate (in days^{-1}) and conjunctival swelling (in pixels) as responses, a fixed four-level factor of measurement point (i.e. dpi) on conjunctival swelling, and a random effect of isolate identity. Since transmission rate is only measured once, residual (within-isolate) variance for this trait is constrained to zero and no residual covariance between traits is modelled. All variance in transmission rate is then partitioned as among-isolate variance, allowing estimation of covariance with the random effects of isolate (C) identity on conjunctival swelling. This analysis yielded a significant positive estimate (SE) of the covariance ($COV_{CTR,SW}$ = 0.86 (0.42); likelihood ratio comparison to a reduced model with no covariance $\chi^2_1 = 4.10$, $p = 0.043$). Scaling by the among-isolate variance in conjunctival swelling yields a linear regression coefficient (SE) of + 0.002 (0.001) infections per day/pixel (i.e. the change in transmission rate as we increase swelling by one pixel).

Determining infection duration for sub-lethal isolates

For those isolates that did not give rise to putative host mortality (i.e. sub-lethal isolates), we estimated infection duration as the duration of the experiment (34 days) + 1 day. Indeed, all sub-lethal isolates save four (i.e. 92% - 22 out of 26 sub-lethal isolates) displayed a decrease

in the number of pathogen cells (Fig. S1A), with 35 days approximating the projected infection duration for those sub-lethal isolates (Fig. S1B).

Fig S1. Number of pathogen cells over the course of the experiment. We show the number of pathogen cells in pooled conjunctival and tracheal swabs obtained at 8, 14, 21 and 28 dpi for: **A,** all sub-lethal isolates (i.e. those that did not give rise to putative host mortality); **B,** sub-lethal isolates that displayed a decreasing number of pathogen cells over the course of the experiment only. Points represent raw (jittered) values; we show the best fit regression lines for each isolate (in **A**) or for the mean (in **B**). Note: jittering causes some isolates to have near 0 counts, but all isolates were detectable in the host for the duration of the experiment (i.e., none of the isolates were cleared during the experiment).

A

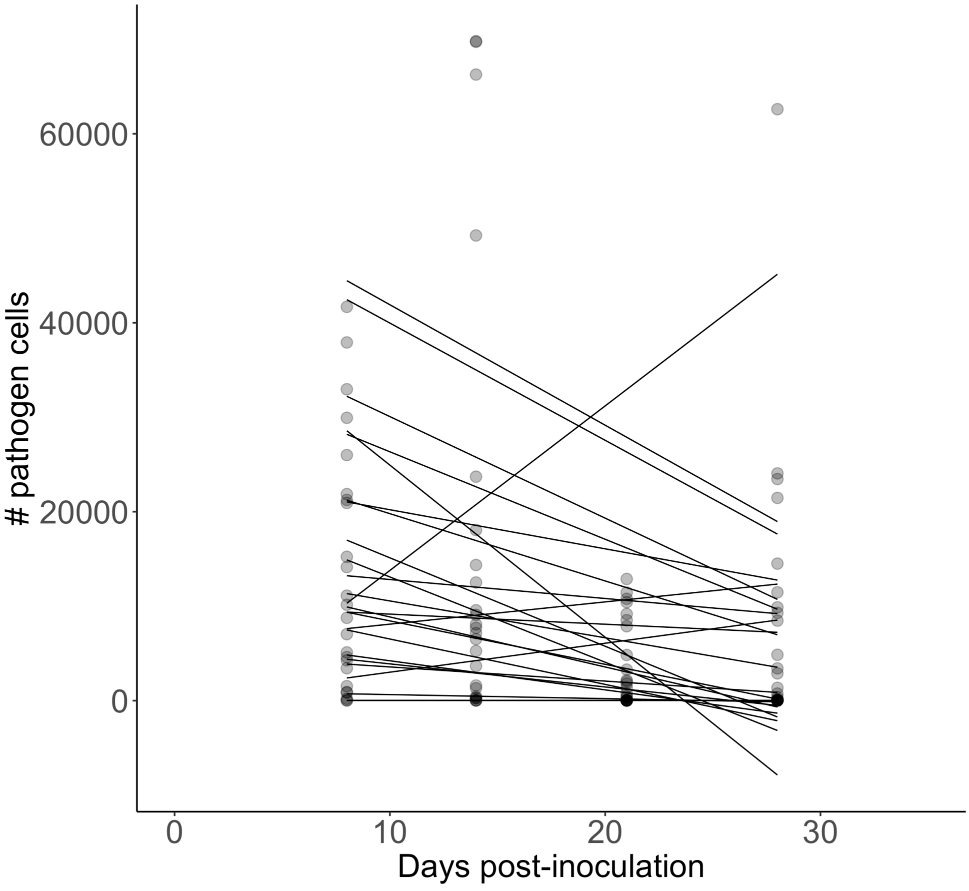

B

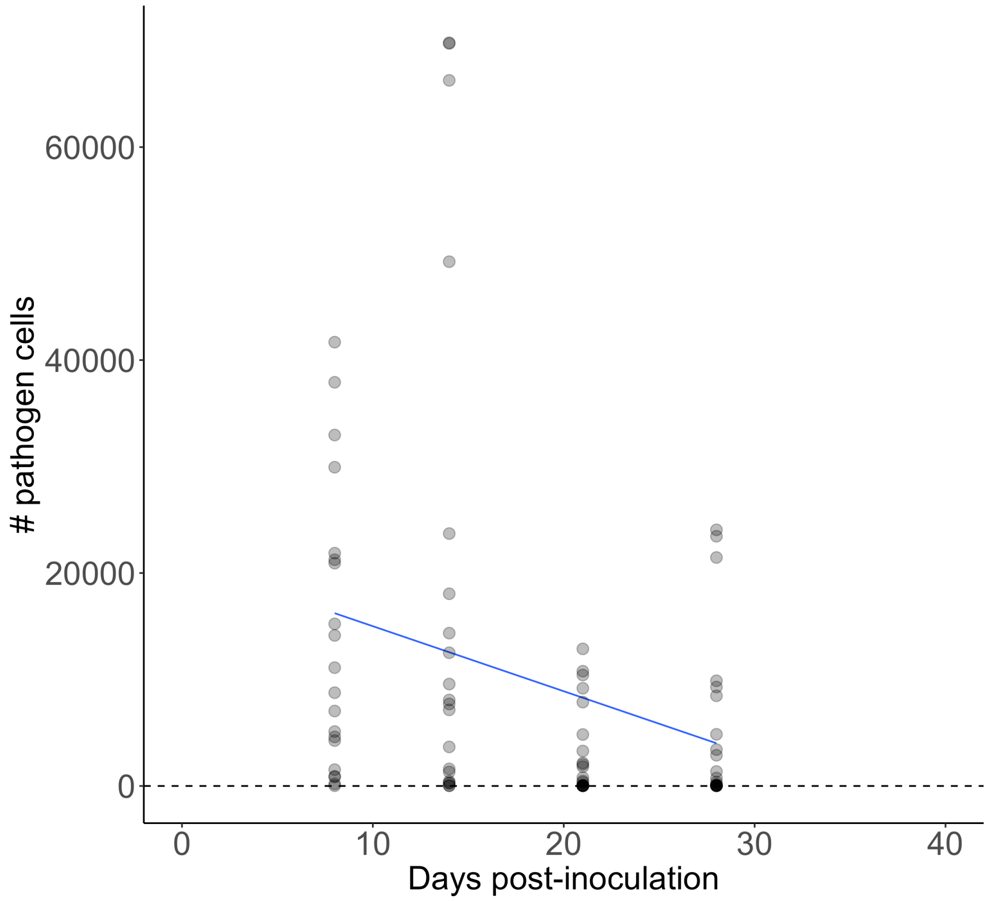

Association between fitness and mean conjunctival swelling

We verified that our estimate of infection duration for sub-lethal isolates (i.e. those that did not cause putative host mortality) did not affect the shape of the relationship between mean conjunctival swelling and fitness, which was bell-shaped when the infection duration of sub-lethal isolates was set to 35 days (see above; Fig. S2A). To do so, we investigated the shape of this relationship in the following two ways. First, we included lethal isolates only (Fig. S2B). Second, based on previous findings that recovery from infection takes between 27 to 83 dpi (Sydenstricker *et al.* 2005), we increased infection duration of those 4 sub-lethal isolates that displayed an increasing number of pathogen cells (see above) to 83 days, with all other sub-lethal isolates remaining at an infection duration of 35 days (Fig. S2C). In both cases, a bell-

shape curve was the best fit line, suggesting that fitness was maximal at an intermediate level of mean conjunctival swelling.

Fig S2. Association between fitness and conjunctival swelling. We show fitness (measured as the product of infection duration and transmission rate to an uninfected sentinel) as a function of mean conjunctival swelling (in pixels) for: **A,** all pathogen isolates, with infection duration of sublethal isolates set to 35 days; **B,** lethal isolates only; **C,** all pathogen isolates, with infection duration of sublethal isolates set either to 35 or to 83 days depending on whether the number of pathogen cells was decreasing or increasing (see Fig. S2). In all cases, the relationship is bell-shaped, as predicted when greater fitness is associated with intermediate values of mean conjunctival swelling. Shapes represent raw values; line are predicted from the models with the standard error represented by the ribbon.

A

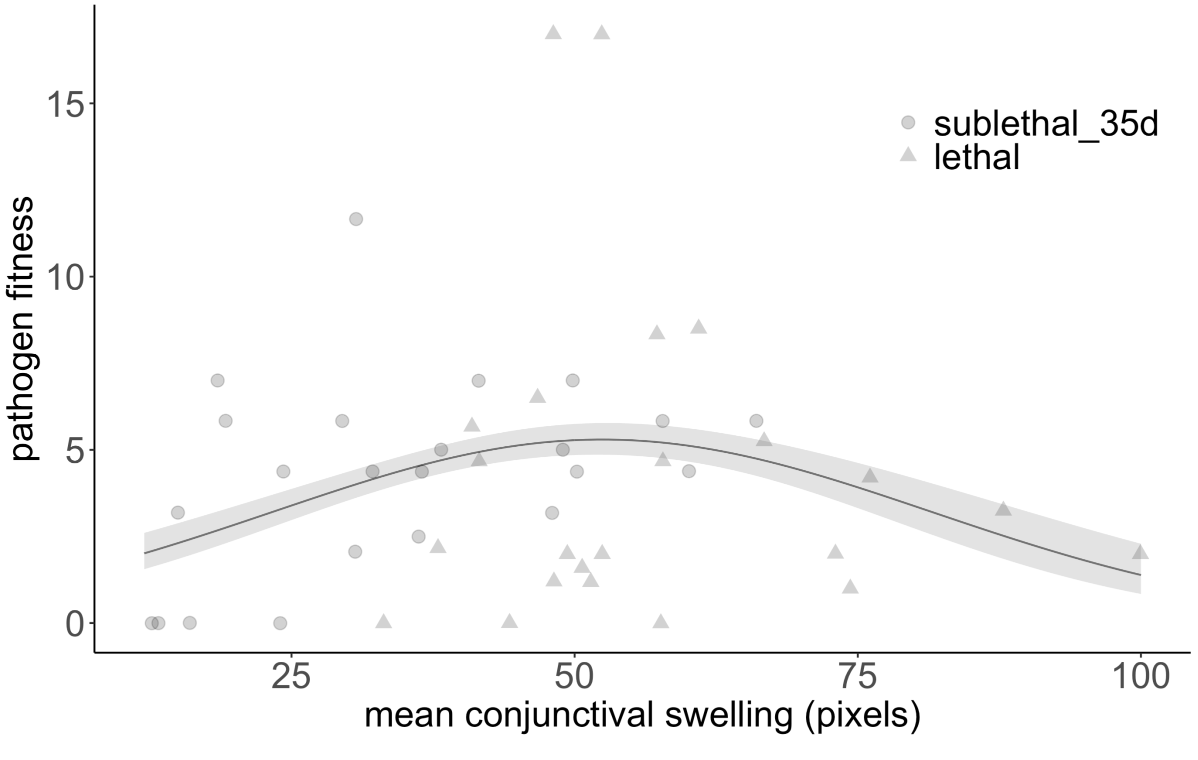

B

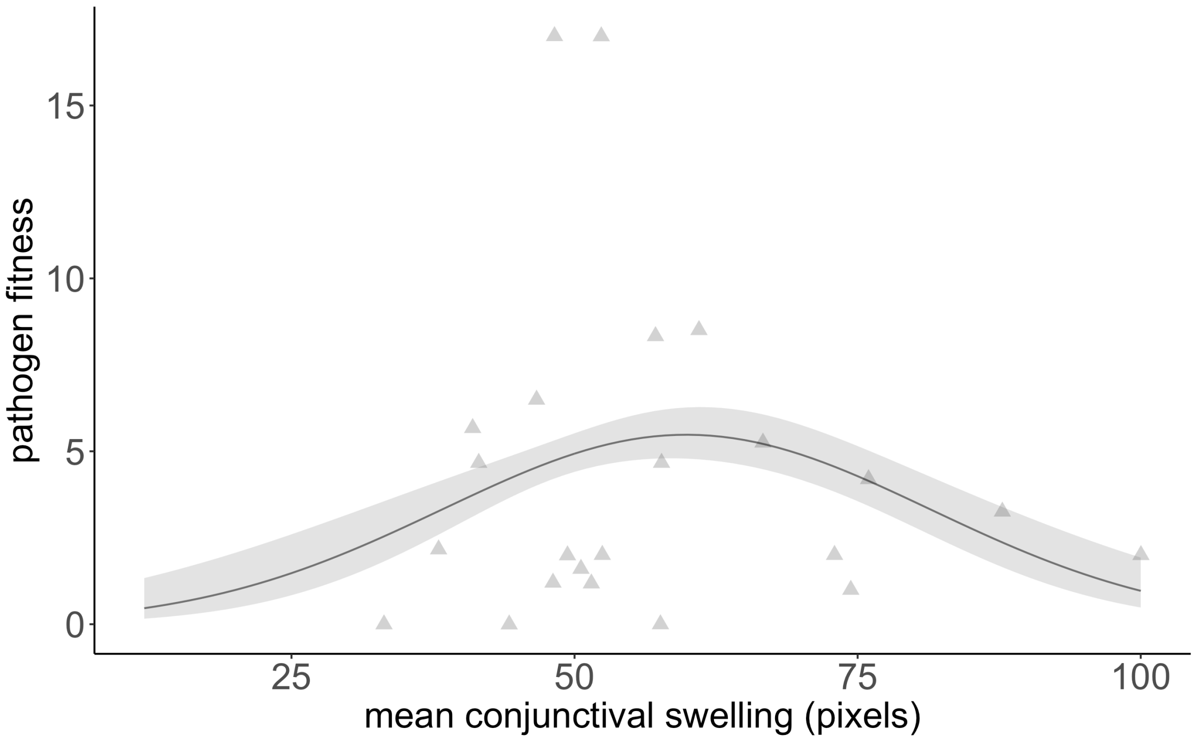

pathogen fitness
15
10
5
0
25
50
75
100
mean conjunctival swelling (pixels)

C

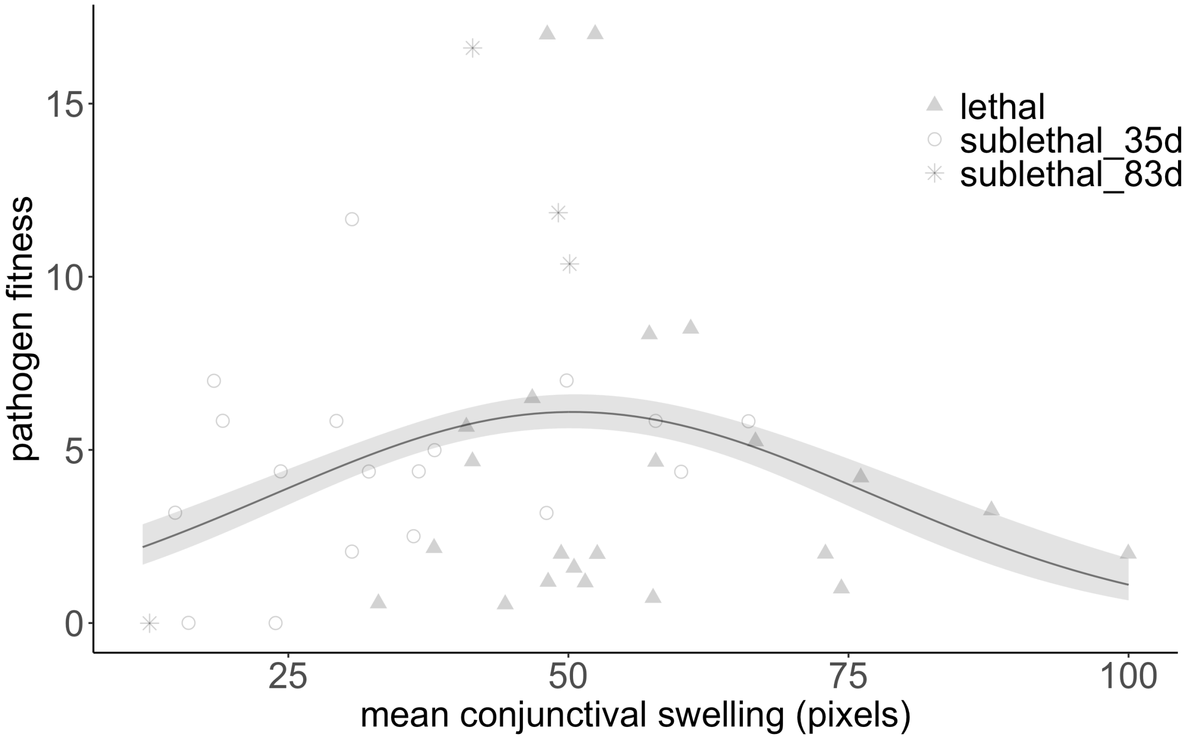

pathogen fitness
15
10
5
0
25
50
75
100
mean conjunctival swelling (pixels)
lethal
sublethal_35d
sublethal_83d

Pathogen load, replication rate and transmission

The peak number of pathogen cells across the 47 successfully established infections during the course of the 34 day-experiment was 45712 ± 93154 bacterial cells/qPCR reaction. Two isolates, however, only managed to achieve a peak number of pathogen cells that were at the absolute lowest limit of assay detection (lower limit of quantification is ~24 copies per assay (Tardy *et al* 2019); note the previously reported limit of 28 copies per assay was a typo). One isolate had a peak number of 23.8 bacterial cells/qPCR reaction, while a second had a peak of 26.3 bacterial cells/qPCR reaction. These values were at least an order of magnitude lower than the peaks attained by all other isolates and were 3 orders of magnitude lower on average. That these peaks are around the lower limit of assay detectability, they clearly showed next to no replication during the experiment. Given that a minimum number of pathogen cells and replication will inevitably be required for transmission, we excluded these two isolates from the analyses and considered the effect of variation in pathogen load and replication rate on transmission rate for the remaining 96% of infections.

METHODS

R code

```
# import data:
EvolLett <- read.csv("Bonneaud et al_EvolLett_2020.csv")
# consider only inoculations that successfully established an infection:
INF<-subset(EvolLett, EvolLett$infection==1)

####1. ASSOCIATION HOST MORTALITY AND TRANSMISSION
INF$day_transm
# we need a rate of transmission = 1/number of days to transmission event:
```

```
INF$ratetrans <- 1/(INF$day_transm)
# we have NA when the sentinel remained uninfected; change it to transmission rate = 0:
INF$ratetrans[is.na(INF$ratetrans)] = 0
hist(INF$ratetrans)
hist(log(INF$ratetrans)) # improve distribution
INF$lratetrans <- log(INF$ratetrans + 0.1) # add small value to get rid of 0 values

# testing the association between virulence and transmission rate:
M1 <- lm(lratetrans ~ mortality,
         data = INF)
summary(M1)

####2. ASSOCIATION MEAN CONJUNCTIVAL SWELLING AND TRANSMISSION
M2 <- lm(lratetrans ~ mean_swell,
           data = INF)
summary(M2)

####3. TESTING THE VIRULENCE FITNESS ASSOCIATION
# Is there stabilizing selection on virulence?
require(mgcv); n <- 100; set.seed(2)
x <- runif(n); y <- x + x^2*.2 + rnorm(n) *.1
M3 <- mgcv::gam(fitness ~s(mean_swell)+ mean_swell,
           data=INF,
           method="REML")
summary(M3)
```

```
# when we remove 2 outliers of very high fitness:
INF2<-subset(INF,INF$fitness<15)
M3b <- mgcv::gam(fitness ~s(mean_swell)+ mean_swell,
                data=INF2,
                method="REML")
summary(M3b)

# Is there linear effect of virulence on fitness?
M4 <- lm(fitness~ mean_swell,
      data=INF)
summary(M4)

####4. PATHOGEN LOAD
#2 isolates out of the 47 successfully established infections, maintained levels of pathogen
#load that were at the lower limit of assay detectability throughout the entire experiment,
#indicating that they were not replicating. These 2 isolates were excluded from the analyses
INFRES <-subset(INF, INF$included=='1')

## Peak pathogen load:
hist(INFRES$peak_load)
INFRES$lpeak <- log(INFRES$peak_load)
M5a <- lm(lratetrans ~ lpeak,
            data = INFRES)
summary(M5a)
## Total pathogen load
```

```
hist(INFRES$total_load)
INFRES$ltotal <- log(INFRES$total_load)
M5b<- lm(lratetrans ~ ltotal,
         data = INFRES)
summary(M5b)

## Rate of replication
hist(INFRES$rate_load)
INFRES$lrate <- log(INFRES$rate_load)
M5c <- lm(lratetrans ~ lrate,
         data = INFRES)
summary(M5c)

### Association between transmission rate and virulence with load as explanatory term
M6a <- lm(lratetrans ~ mean_swell + lpeak,
          data = INFRES)
summary(M6a)
M6b <- lm(lratetrans ~ mean_swell + ltotal,
          data = INFRES)
summary(M6b)
M6c <- lm(lratetrans ~ mean_swell + lrate,
          data = INFRES)
summary(M6c)
```

References

Butler, D.G., Cullis, B.R., Gilmour, A.R., Gogel, B.G. & Thompson, R. (2017). ASReml-R Reference Manual. VSN International Ltd Hemel Hempstead, HP1 1ES, UK.

Sydenstricker, K.V., Dhondt, A.A., Ley, D.H. & Kollias, G.V. (2005). Re-exposure of captive house finches that recovered from Mycoplasma gallisepticum infection. *Journal of Wildlife Diseases*, 41, 326-333.

Tardy, L., Giraudeau, M., Hill, G.E., McGraw, K.J. & Bonneaud, C. (2019). Contrasting evolution of virulence and replication rate in an emerging bacterial pathogen. *Proc. Natl. Acad. Sci. U. S. A.*
